# Precursors to Systemic Sclerosis and Systemic Lupus Erythematosus: From Undifferentiated Connective Tissue Disease to the Development of Identifiable Connective Tissue Diseases

**DOI:** 10.3389/fimmu.2022.869172

**Published:** 2022-05-05

**Authors:** Leonardo Martin Calderon, Janet E. Pope

**Affiliations:** ^1^Department of Medicine, Schulich School of Medicine and Dentistry, University of Western Ontario, London, ON, Canada; ^2^Division of Rheumatology, St. Joseph’s Health Care, Schulich School of Medicine and Dentistry, University of Western Ontario, London, ON, Canada

**Keywords:** systemic sclerosis, scleroderma, prescleroderma, pathogenesis, innate immunity, adaptive immunity, systemic lupus erythematosus, autoimmunity

## Abstract

The pathogenesis of connective tissue diseases (CTDs), such as systemic lupus erythematosus (SLE) and systemic sclerosis (SSc), is characterized by derangements of the innate and adaptive immune system, and inflammatory pathways leading to autoimmunity, chronic cytokine production, and chronic inflammation. The diagnosis of these diseases is based on meeting established criteria with symptoms, signs and autoantibodies. However, there are pre-clinical states where criteria are not fulfilled but biochemical and autoimmune derangements are present. Understanding the underlying processes responsible for disease pathogenesis in pre-clinical states, which place patients at increased risk for the development of established connective tissue diseases, represents an opportunity for early identification and potentially enables timely treatment with the goal of limiting disease progression and improved prognosis. This scoping review describes the role of the innate and adaptive immune responses in the pre-clinical states of undifferentiated CTD at risk for SSc and prescleroderma, the evolution of antibodies from nonspecific to specific antinuclear antibodies prior to SLE development, and the signaling pathways and inflammatory markers of fibroblast, endothelial, and T cell activation underlying immune dysregulation in these pre-clinical states.

## Introduction

Systemic sclerosis (SSc) is a rare multisystem autoimmune connective tissue disease (CTD) characterized by fibrosis of the skin and internal organs, vasculopathy, and autoimmunity with distinct antibodies. SSc is classified using the American College of Rheumatology/European League of Rheumatism (ACR/EULAR) 2013 criteria (1). However, there are pre-morbid clinical states, including Undifferentiated Connective Tissue Disease at risk for Systemic Sclerosis (UCTD-risk-SSc) and prescleroderma, where autoimmunity and dysregulation of inflammatory pathways occur without the presence of clinical symptoms (2). UCTD-risk-SSc, also known as very early/early SSc, is a label given to patients who do not meet the ACR/EULAR 2013 criteria, but who present with Raynaud’s Phenomenon (RP) and either typical SSc capillaroscopic findings (megacapillaries or avascular areas) or serum marker antibodies (anti-centromere, anti-topoisomerase I, anti-RNA polymerase III, anti-Th/To, and anti-Pm-Scl) (3, 4). UCTD-risk-SSc patients have a 35-79% risk of developing definite SSc over time (5–7). Prescleroderma is diagnosed in patients with RP who present with serum marker autoantibodies (anti-centromere or anti-topoisomerase I) and immunofluorescence derived antinuclear antibodies (ANA) at titre >1:320 or serum antibodies and avascular capillaroscopic changes or ANA positivity at 1:320 and avascular areas (7). Moreover, patients with prescleroderma have an even higher risk of developing established SSc than UCTD-risk-SSc (7). Making a diagnosis and intervening early may change the trajectory of disease in these patients.

Another CTD with pre-clinical stages progressing to identifiable disease is systemic lupus erythematous (SLE). SLE which is characterized by features such as arthritis, rash, photosensitivity, serositis, cytopenias, mucositis, glomerulonephritis, fevers and fatigue, may onset insidiously and can be difficult to differentiate from other autoimmune diseases initially (8, 9). Commonly ANA will pre-date SLE diagnosis by years during undifferentiated pre-clinical stages termed “incomplete SLE” or “possible SLE” when ACR criteria for SLE are not met (10, 11). Approximately 55% of patients with incomplete SLE (iSLE) develop SLE (12). Furthermore, as disease progression occurs, more specific antibodies for SLE are produced such as anti-double stranded DNA and anti-Smith antibodies (10, 13).

Ultimately, the changes observed in these pre-clinical stages with varying likelihood of progression to full-blown disease are insidious and driven by derangements in inflammatory signalling and autoimmunity. The purpose of our scoping review was to elucidate the role of the innate and adaptive immune systems and dysregulated signaling pathways in pre-clinical states, and their contribution to the establishment of full-blown disease.

## Search Strategy

**Figure 1:**
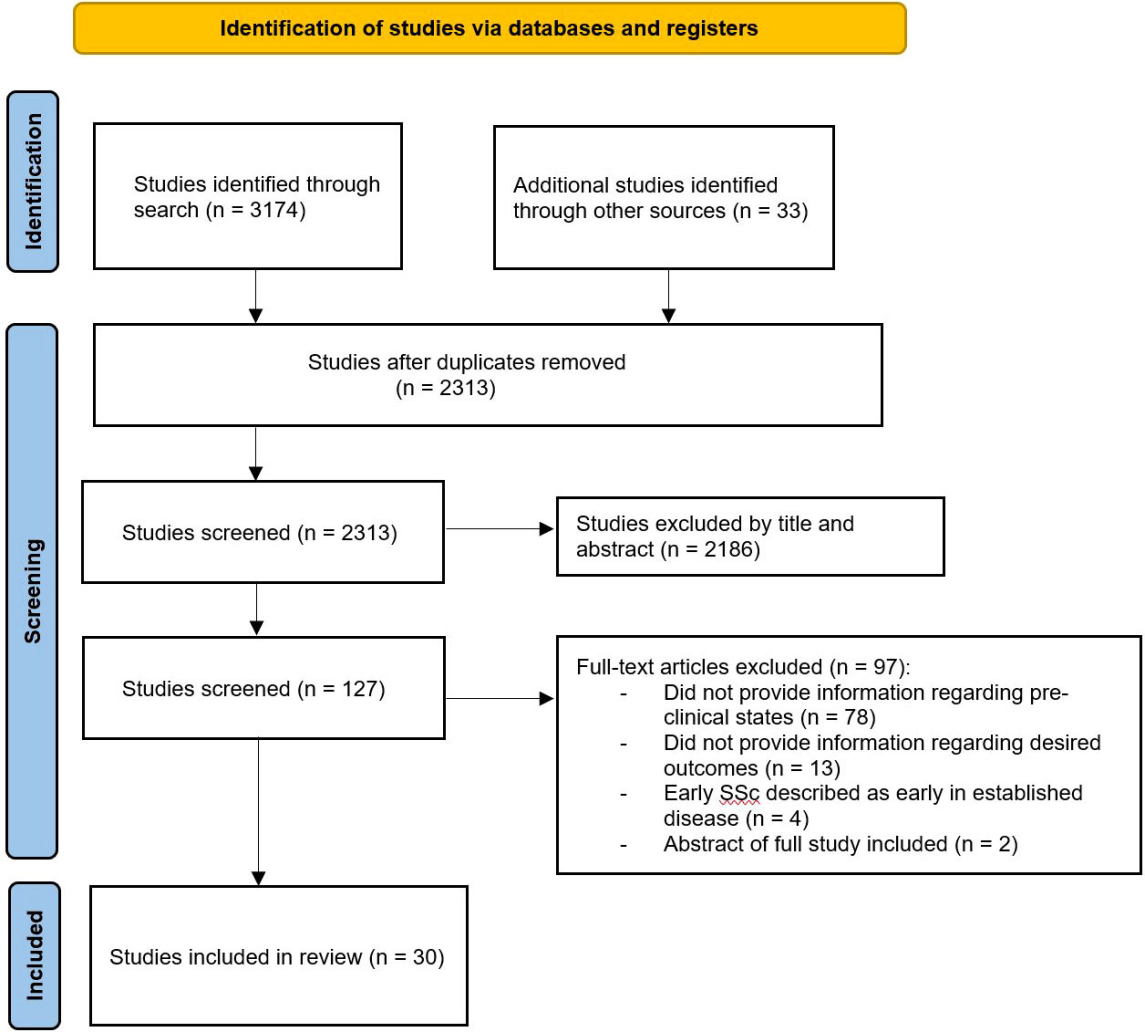
Flow diagram of scoping review selection process.

Our search strategy was developed with an experienced information specialist (Supplementary Material). We searched the databases EMBASE and MEDLINE with restrictions for the English language and included peer-reviewed manuscripts as well as conference abstracts. We sought to include studies which provided information regarding the role of adaptive and innate immune systems and the dysregulation of pathways which contributed to the development of classifiable SSc or SLE. Therefore, we included studies which explicitly studied individuals termed as UCTD-risk-SSc, Very early/early SSc, prescleroderma, pre-SLE, incomplete SLE, or lupus-like. Studies were excluded if they provided information regarding inflammatory pathways where patients with established disease were investigated. The search and inclusion of studies was performed by one reviewer (LMC) with review of included studies performed by both authors (LMC & JEP). Our search yielded 2313 manuscripts after duplicates were removed on August 10, 2021 and pertinent manuscripts have been included (**Figure 1**).

## Systemic Sclerosis

### Dysregulated Signalling Pathways and Autoimmunity

Progressive inflammation, vasculopathy and fibrosis orchestrated by aberrant cytokine production is a hallmark of SSc. Chemokines involved in extracellular matrix deposition, erroneous activation of fibroblasts, and anomalous immune system activation, including CCL2, MIP-1α/CCL3, CCL4, CCL7/MCP-3, and CXCL8, have been observed to be significantly upregulated in the serum of established SSc patients when compared to healthy controls (14–16). However, the presence of these chemokines is more nuanced in pre-clinical disease. Vettori et. al., compared the serum of UCTD-risk-SSc patients to fibromyalgia and/or osteoarthritis controls without RP, and definite SSc patients for soluble intercellular adhesion molecule-1 (sICAM-1), soluble vascular adhesion molecule-1 (sVCAM-1), CCL2, CXCL8, IL-13, IL-33, and transforming growth factor-β (TGF-β) (17). A significant increase was observed in sICAM-1, CCL2, CXCL8, and IL-13 along a disease spectrum gradient from UCTD-risk-SSc to limited cutaneous SSc (lcSSc) to diffuse cutaneous SSc (dcSSc). sICAM-1 is involved in the transmigration of leukocytes from vessels to endothelium and promotes inflammation through T cell activation and cytokine production (18, 19). CXCL8 and CCL2 are pro-fibrotic alter angiogenesis, and affect the migration of monocytes, T cells, and neutrophils (20–22). IL-13 contributes to fibrogenesis through fibroblast activation and TGF-β stimulation (23). Consequently, chemokines increase as disease severity worsens highlighting the progressive derangement of vasculature and autoimmune changes in SSc. Interestingly, higher IL-33 levels were found in UCTD-risk-SSc patients compared to controls and established SSc. IL-33 induces IL-4, IL-5, and IL-13 production leading to arterial vessel media hypertrophy and eosinophilic and mononuclear cell infiltration (24). Therefore, IL-33 functions as a very early mediator in the progression to established SSc, is involved in the fibrotic stage of SSc through IL-13 stimulation; and serves as a predictive marker to elucidate which patients will develop established disease (25).

Other cytokines are abnormal in UCTD-risk-SSc including soluble IL-2 receptor alpha (sIL-2Rα), aminoterminal propeptide of type III collagen (PIIINP), and CXCL4 (7, 26, 27). sIL-2Rα functions as a marker of T-cell activation, whereas PIIINP functions as a marker of collagen formation and fibroblast activation (28, 29). CXCL4 functions as a potent anti-angiogenic chemokine and serves to inhibit endothelial cell proliferation and migration (30). Additionally, CXCL4 has pro-fibrotic capabilities through inhibiting interferon-gamma (IFN-γ) expression and stimulating IL-13 and IL-4 production (31). CXCL4 levels, measured from serum, were higher in UCTD-risk-SSc than controls and were associated with anti-Scl 70 antibodies and sICAM-1 (27, 32). Furthermore, CXCL4 levels, drawn from non-platelet poor plasma, were reported to correlate with extent of skin fibrosis and were predictive of pulmonary arterial hypertension and lung and skin fibrosis progression in SSc (33).

Type I IFN represents another significant contributor to the pathogenesis of SSc through the upregulation of genes involved in the activation of the innate and adaptive immune systems. The increased expression of these type I IFN regulated genes, termed the type I IFN signature, has been previously observed in SLE and other autoimmune diseases (34, 35). Brkic et al., investigated the whole-blood samples of healthy controls without RP, patients with primary RP, UCTD-risk-SSc, and definite SSc patients to determine the expression of 11 type I IFN inducible genes (36). Authors report increased type I IFN related gene expression in UCTD-risk-SSc patients compared to healthy controls, but not in primary RP compared to controls. This finding eludes to the early contribution of the type I IFN pathway in the pathogenesis of SSc. Furthermore, the presence of polymorphisms of IFN regulated genes have been found to confer increased risk of SSc (37).

### Vasculopathy and Fibrogenesis

Cossu et al. investigated angiogenetic and endothelial dysfunction markers involved in vasculopathy (38). Authors sampled the serum of healthy controls without RP, UCTD-risk-SSc, lcSSc, and dcSSc patients for angiopoietin-2 (ang-2), CXCL16, e-selectin, sICAM-1, CXCL8, sVCAM-1, and VEGF. There was a significant trend along a disease spectrum from controls to UCTD-risk-SSc to lcSSc and to dcSSc for ang-2, CXCL16, e-selectin, and sICAM-1. Authors also observed a significant difference in ang-2 between controls and UCTD-risk-SSc. Ang-2’s functioning is contextual as it facilitates angiogenesis if VEGF is present, but causes blood vessel regression if pro-angiogenic stimuli are absent (35). Clinically, ang-2 correlates with the extent of skin involvement in SSc as measured by the modified Rodnan skin score (mRSS), disease activity, and C-reactive protein (39). Tabata et. al., found that IGF-1, VEGF, and RANTES levels are significantly higher in mild established SSc compared to pre-clinical SSc (40).

Fibrogenic inflammatory pathways resulting from chronic inflammation and orchestrated through fibroblast dysfunction lead to excessive accumulation of extracellular matrix components, including hyaluronic acid, fibronectin, and proteoglycans, in SSc (41). Sera of healthy controls without RP, UCTD-risk-SSc, and non-fibrotic SSc patients were analyzed whereby elevated markers (CXCL10/IP-10, CXCL11/I-TAC, tumor necrosis factor receptor type II (TNFRII), and chitinase 3-like protein 1) were higher in UCTD-risk-SSc patients compared to controls (42). CXCL10 and CXCL11 are angiostatic and migration chemokines which drive smooth muscle cell proliferation, and recruit T cells, monocytes, and natural killer cells (43–45). Importantly, CXCL10 and CXCL11 levels are associated with UCTD-risk-SSc patients most at risk for developing established SSc (25, 46). Furthermore, CXCL10 and CXCL11 are observed to be correlated with type I IFN signature and decrease with type I IFN receptor blockade with anifrolumab (47).TNFRII has a role in the proliferation and activation of regulatory T cells (48). Additionally, TNFRII co-stimulated lymphocytes secrete pro-fibrotic cytokines in patients with SSc (49). Chitinase 3-like protein 1 has been implicated in regulating and stimulating angiogenesis and fibrogenesis through activation of Syndencan-1 and focal adhesion kinase (50). Furthermore, in SSc patients, chitinase 3-like protein 1 has been correlated with articular involvement and T cell activation (51). These findings highlight the interplay between the adaptive and innate immune systems alongside fibrogenesis.

**Figure 2:**
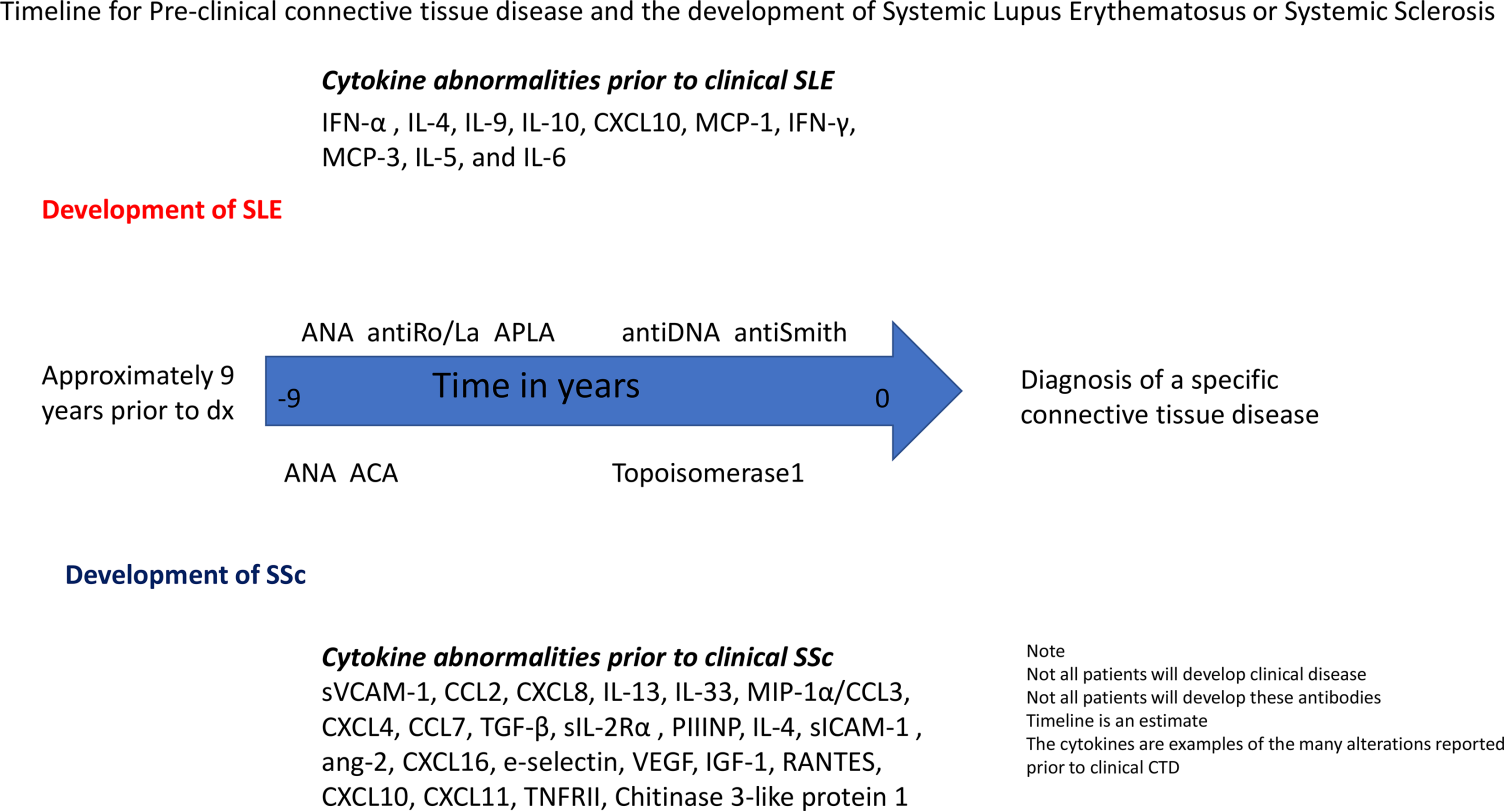
Timeline for Pre-clinical connective tissue disease and the development of Systemic Lupus Erythematosus or Systemic Sclerosis. Legend: SLE, Systemic lupus erythematosus; SSc, Systemic sclerosis; ANA, antinuclear antibody; APLA, antiphospholipid antibodies; ACA, anticentromere antibodies.

Alterations of natural killer (CD 56+) and natural killer T cells (CD56+ CD3+) in early SSc compared to controls, primary RP, and established SSc were found and thought to be related to differential Toll-like receptor (TLR) 1/2 stimulation (52). Early SSc demonstrated an intermediate activation pattern regarding CD56+ secretion of IL-6, TNF-a, and MIP-1α/CCL3 compared to controls with significant differences of IL-6 secretion. An increasing trend in CD56+ activation for TNF-α and CCL3 occurred between early SSc and controls. This pattern of elevated IL-6, TNF-α, and CCL3 alludes to the role of underlying innate immune mechanisms in prescleroderma or early SSc; which, may eventually lead to established SSc. The development of SSc is shown over time (**Figure 2**).

## Systemic Erythematosus Lupus

### Autoimmunity and Dysregulated Pathways

Antibodies predate the diagnosis of SLE by multiple years in a characteristic pattern evolving from non-specific ANA to more specific SLE antibodies prior to diagnosis. In a large serology study, a cohort of 130 military personnel who ultimately developed SLE were followed from first detection of ANA to diagnosis of SLE a median of 9.2 years later (11). Furthermore, anti-Ro, anti-La, anti-phospholipid, anti-double stranded DNA, anti-Smith, and anti-nuclear ribonucleoprotein (anti-RNP) antibodies were reported to have a time of first detection to diagnosis of 9.4 years, 8.1 years, 7.6 years, 9.3 years, 8.1 years, and 7.2 years, respectively. This observed pattern, corroborated by further studies, reflects progressive antibody evolution towards more specific SLE antibodies over time in patients ultimately diagnosed with SLE as ANA, anti-double stranded DNA, and anti-Smith antibodies have 86%, 94.7%, and 99% specificity, respectively (53–56). The presence and development of SLE specific antibodies can also serve as predictive makers of developing established disease. Munroe et. al., investigated unaffected blood relatives of SLE patients to identify risk factors of disease establishment (57). Relatives who developed SLE had elevated ANA and anti-Ro titers, and were likely to be anti-dsDNA and anti-RNP positive at baseline and follow up compared to those who did not transition. Anti-cardiolipin antibody positive patients also had more risk of developing SLE (58–60).

Cytokine changes in pre-clinical SLE have been studied (61). Interferon-α, IL-4, IL-9, IL-10, CXCL10 and monocyte chemotactic protein-1 (MCP-1/CCL2) were studied in sera of 35 patients prior to established SLE. CXCL10 was significantly higher in pre-clinical sera compared to controls and was correlated with interferon-α. One of the drivers of innate and adaptive immune dysregulation occurs through an up-regulation of interferon regulated genes, which is also known as the IFN signature of SLE (62). IFN-α, a type I IFN, stimulation leads to increased dendritic cell maturation, increased Th1 cell development and response, and enhanced NK, B, and T cell proliferation and survival (62). IFN-α correlates positively with IgG, and negatively with IgM autoantibodies (63). CXCL10 and IFN-α concentrations are higher in pre-clinical patients who are positive for any antibody compared to antibody negative patients.

Type II IFN (IFN-γ) is additionally implicated in SLE development (64). IFN-γ leads to production of IFN-α and the B-lymphocyte stimulator (BLyS) (65, 66). BLyS, otherwise known as B-cell activating factor of the TNF family (BAFF), is produced by innate immune cells and serves as a mediator of B cell proliferation and survival (67–69). BLyS induces a Th1 cellular response which coordinates both innate and cytotoxic immunity (65). Munroe et al., studied the timing and role of type I and II IFN, IFN-associated mediators, and antibody formation in pre-clinical patients who would later develop established SLE (70). Elevated IFN-γ, CXCL10, and MCP-3 levels occurred prior to IFN-α activity and antibodies. IFN-γ and MCP-3 are abnormal more than 4 years prior to the development of SLE. Therefore, though type I IFN is observed to be elevated in association with antibody positivity prior to SLE diagnosis, type II IFN and IFN-associated mediators seem to represent the pathogenetic intermediaries altering innate and adaptive immune system derangements through elevation of IFN-α and autoantibody formation. These findings agree with a finding that IFN-γ, IL-5, and IL-6 were elevated at least 3.5 years prior to classification (71). Importantly, these observed temporal differences may be secondary to the measurement techniques used in these studies and further investigations with direct measurements tools, such as single-molecule arrays, may further elucidate the temporal relationship between type I and II IFN. **Figure 2** shows a timeline for the development of SLE and SSc.

## Discussion

Understanding the immunological and inflammatory perturbations involved in the development of CTDs such as SSc and SLE provides clinicians with an opportunity to recognize pre-clinical patients that may benefit from close monitoring, investigations, and potentially early intervention to limit disease progression. Pre-clinical disease states, such as UCTD-risk-SSc, prescleroderma, and incomplete SLE, present with underlying aberrations, often years before clinical disease is present, of the innate and adaptive immune systems, and inflammatory pathways which drive pathogenesis and increase risk of developing established disease.

The pathogenic mechanisms present in UCTD-risk-SSc and prescleroderma include immune signal dysregulations, erroneous immune system recruitment, aberrant angiogenesis leading to vasculopathy, and inappropriate fibroblast activation leading to tissue fibrosis. Multiple cytokines are observed to increase along a disease spectrum from UCTD-risk-SSc to classified SSc and include sICAM-1, CCL2, CXCL8, ang-2, CXCL16, e-selectin, and IL-13 (**Table 1**). The mechanism of action of these cytokines includes transmigration of lymphocytes endothelium, innate immune cell activation and signal propagation, and extracellular matrix deposition. Furthermore, there are disease markers which are observed to be predictive of SSc and include sIL-2Rα, PIIINP, CXCL4, CXCL10, and CXCL11 (**Table 2**). Patients with SSc who have the limited cutaneous SSc subset frequently develop RP and anti-centromere antibody 8 years before other manifestations of SSc often followed by dilated nailfold capillaries, then puffy fingers or sclerodactyly and other features of SSc (2–4). The presence or absence of these features is significant in risk stratification where patients with RP but without antibodies or nailfold capillary changes are at 1.8% risk of definite SSc compared to 79.5% in those with RP and positive antibodies and nailfold capillary changes (5). At this point in time, other than treating RP to try to prevent ischemic changes, there is no specific treatment to change the natural history of future development of SSc. Also, 1/3 may develop SSc over the next 5 years (so 2/3 won’t) and this can lead to over-diagnosis, and patient anxiety. Interventions such as smoking cessation and reducing RP attacks and encouraging a healthy lifestyle including a diet high in omega3 fatty acids may be appropriate but this is speculation. Patients with diffuse cutaneous SSc do not develop RP until close to their diagnosis (often 1 to 2 years before or at the time of other signs and symptoms of SSc), so finding prescleroderma clinical features in the majority of these patients has not been possible.

**Table 1:** Elevated chemokines observed in UCTD-risk-SSC orchestrating SSc pathogenesis.

Cytkine	Function
sICAM-1	Transmigration of leukocytes, T cells activation
CCL2	Chemotaxis of monocytes, T cells, neutrophils
CXCL8	Angiogenesis induction, immune cell proliferation
IL-13	Fibroblast activation, TGF-β secretion stimulation
Ang-2	Angiogenesis induction, monocyte activation
TNFRII	Regulatory T cell proliferation, profibrotic cytokine secretion
CHI3L1/YKL-40	Angiogenesis and fibrogenesis regulation

**Table 2:** Taxa Cytokines observed to be predictors of SSc development.

Cytkine	Function
sIL-2Rα	Marker of T cell activation and proliferation
PIIINP	Marker of collagen formation and fibroblast activation
CXCL4	IL-13 and IL-4 stimulation
CXCL10	Smooth muscle cell proliferation, immune cell chemotaxis
CXCL11	T cell, monocyte, natural killer recruitment

Likewise, SLE development is rooted in aberrations of the innate and adaptive immune systems. Pre-clinical SLE is characterized by an evolving IFN signature and progressive SLE-specific antibody formation prior to disease classification. IFN-γ and IFN associated mediators can predate diagnoses by 3.5 years, and are present prior to and alongside antibody positivity. Throughout pre-clinical SLE, antibody formation occurs in a pattern that evolves from non-specific ANA to more specific SLE antibodies. Namely, ANA and anti-Ro formation can predate diagnosis by 9 years or more but are considered less specific. Whereas, the more specific anti-Smith and anti-dsDNA develop closer to disease onset. The development of SLE specific antibodies can function as predictive markers of transformation to clinical SLE.

Clinically, it is difficult to ascertain what to do with the findings. Other than close monitoring of patients at risk, it is not feasible to check cytokine panels (with high variability) and redoing antibodies is likely not cost effective. However, the changes in immune regulation that predate clinical CTD help in the understanding of pathogenesis and may in future provide targeted treatment for patients with a high probability of converting to chronic debilitating disease. It has been suggested that treating patients at risk for SLE with hydroxychloroquine may change the disease trajectory but large controlled studies are needed to determine if there is benefit in this approach (72); one such multicenter, randomized, placebo-controlled, double-blind clinical trial is currently underway (NCT0303118). Interestingly, there are already drug targets in clinically active SLE targeting signalling that has been shown to be abnormal prior to disease onset such as BlyS (belimumab) and type I interferon with anifrolumab. Intervening prior to clinical disease would not be appropriate with the knowledge we have but in future, personalized medicine may help to give a more robust prediction of who will develop chronic autoimmune CTD.

## Conclusion

Ultimately, the coordinated dysregulation of the innate and adaptive immune systems, and inflammatory signalling pathways leads to the pathogenesis of connective tissue disease. Our improved understanding of these underlying aberrations in pre-clinical stages of disease will serve to better identify patients at increased risk.

## Author Contributions

LM and JP were involved in study design, review of literature, and manuscript writing. All authors reviewed the manuscript and approved the final version.

## Conflict of Interest

The authors declare that the research was conducted in the absence of any commercial or financial relationships that could be construed as a potential conflict of interest.

## Publisher’s Note

All claims expressed in this article are solely those of the authors and do not necessarily represent those of their affiliated organizations, or those of the publisher, the editors and the reviewers. Any product that may be evaluated in this article, or claim that may be made by its manufacturer, is not guaranteed or endorsed by the publisher.

## References

[1] van den Hoogen F Khanna D Fransen J Johnson SR Baron M Tyndall A. Classification Criteria for Systemic Sclerosis: An American College of Rheumatology/European League Against Rheumatism Collaborative Initiative. Ann Rheum Dis (2013) 72(11):1747–55. doi: 10.1136/annrheumdis-2013-204424 24092682

[2] Avouac J Fransen J Walker UA Riccieri V Smith V Muller C. Preliminary Criteria for the Very Early Diagnosis of Systemic Sclerosis: Results of a Delphi Consensus Study From EULAR Scleroderma Trials and Research Group. Ann Rheum Dis (2011) 70(3):476–81. doi: 10.1136/ard.2010.136929 21081523

[3] Valentini G. Undifferentiated Connective Tissue Disease at Risk for Systemic Sclerosis (SSc) (So Far Referred to as Very Early/Early SSc or Pre-SSc). Autoimmun Rev (2015) 14(3):210–3. doi: 10.1016/j.autrev.2014.11.002 25461837

[4] Matucci-Cerinic M Bellando-Randone S Lepri G Bruni C Guiducci S. *Very Early* Versus *Early* Disease: The Evolving Definition of the ‘*Many Faces*’ of Systemic Sclerosis. Ann Rheum Dis (2013) 72(3):319–21. doi: 10.1136/annrheumdis-2012-202295 23178210

[5] Koenig M Joyal F Fritzler MJ Roussin A Abrahamowicz M Boire G. Autoantibodies and Microvascular Damage are Independent Predictive Factors for the Progression of Raynaud’s Phenomenon to Systemic Sclerosis: A Twenty-Year Prospective Study of 586 Patients, With Validation of Proposed Criteria for Early Systemic Sclerosis. Arthritis Rheumatol (2008) 58(12):3902–12. doi: 10.1002/art.24038 19035499

[6] Valentini G Marcoccia A Cuomo G Vettori S Iudici M Bondanini F. Early Systemic Sclerosis: Analysis of the Disease Course in Patients With Marker Autoantibody and/or Capillaroscopic Positivity. Arthritis Care Res (Hoboken) (2014) 66(10):1520–7. doi: 10.1002/acr.22304 24515450

[7] Valentini G Pope JE. Undifferentiated Connective Tissue Disease at Risk for Systemic Sclerosis: Which Patients Might be Labeled Prescleroderma? Autoimmun Rev (2020) 19(11):102659. doi: 10.1016/j.autrev.2020.102659 32942034

[8] Kiriakidou M Ching CL. Systemic Lupus Erythematosus. Ann Intern Med (2020) 172(11):ITC81–96. doi: 10.7326/AITC202006020 32479157

[9] Mosca M Costenbader KH Johnson SR Lorenzoni V Sebastiani GD Hoyer BF. Brief Report: How Do Patients With Newly Diagnosed Systemic Lupus Erythematosus Present? A Multicenter Cohort of Early Systemic Lupus Erythematosus to Inform the Development of New Classification Criteria. Arthritis Rheumatol (2019) 71(1):91–8. doi: 10.1002/art.40674 30035365

[10] Lambers WM Westra J Bootsma H de Leeuw K. From Incomplete to Complete Systemic Lupus Erythematosus; A Review of the Predictive Serological Immune Markers. Semin Arthritis Rheumatol (2021) 51(1):43–8. doi: 10.1016/j.semarthrit.2020.11.006 33360229

[11] Arbuckle MR McClain MT Rubertone MV Scofield RH Dennis GJ James JA. Development of Autoantibodies Before the Clinical Onset of Systemic Lupus Erythematosus. N Engl J Med (2003) 349(16):1526–33. doi: 10.1056/NEJMoa021933 14561795

[12] Lambers WM Westra J Jonkman MF Bootsma H de Leeuw K. Incomplete Systemic Lupus Erythematosus: What Remains After Application of American College of Rheumatology and Systemic Lupus International Collaborating Clinics Criteria? Arthritis Care Res (Hoboken) (2020) 72(5):607–14. doi: 10.1002/acr.23894 PMC7217202 30932354

[13] Eriksson C Kokkonen H Johansson M Hallmans G Wadell G Rantapää-Dahlqvist S. Autoantibodies Predate the Onset of Systemic Lupus Erythematosus in Northern Sweden. Arthritis Res Ther (2011) 13(1):R30. doi: 10.1186/ar3258 21342502 PMC3241374

[14] Codullo V Baldwin HM Singh MD Fraser AR Wilson C Gilmour A. An Investigation of the Inflammatory Cytokine and Chemokine Network in Systemic Sclerosis. Ann Rheum Dis (2011) 70(6):1115–21. doi: 10.1136/ard.2010.137349 21285114

[15] Distler JHW Jüngel A Caretto D Schulze-Horsel U Kowal-Bielecka O Gay RE. Monocyte Chemoattractant Protein 1 Released From Glycosaminoglycans Mediates its Profibrotic Effects in Systemic Sclerosis *via* the Release of Interleukin-4 From T Cells. Arthritis Rheumatol (2006) 54(1):214–25. doi: 10.1002/art.21497 16385517

[16] Yanaba K Komura K Kodera M Matsushita T Hasegawa M Takehara K. Serum Levels of Monocyte Chemotactic Protein-3/CCL7 are Raised in Patients With Systemic Sclerosis: Association With Extent of Skin Sclerosis and Severity of Pulmonary Fibrosis. Ann Rheum Dis (2006) 65(1):124–6. doi: 10.1136/ard.2005.040782 PMC1797996 16344498

[17] Vettori S Cuomo G Iudici M D’Abrosca V Giacco V Barra G. Early Systemic Sclerosis: Serum Profiling of Factors Involved in Endothelial, T-Cell, and Fibroblast Interplay is Marked by Elevated Interleukin-33 Levels. J Clin Immunol (2014) 34(6):663–8. doi: 10.1007/s10875-014-0037-0 24760110

[18] McCabe SM Riddle L Nakamura GR Prashad H Mehta A Berman PW. sICAM-1 Enhances Cytokine Production Stimulated by Alloantigen. Cell Immunol (1993) 150(2):364–75. doi: 10.1006/cimm.1993.1204 8103708

[19] Lawson C Wolf S. ICAM-1 Signaling in Endothelial Cells. Pharmacol Rep (2009) 61(1):22–32. doi: 10.1016/S1734-1140(09)70004-0 19307690

[20] Russo RC Garcia CC Teixeira MM Amaral FA. The CXCL8/IL-8 Chemokine Family and its Receptors in Inflammatory Diseases. Expert Rev Clin Immunol (2014) 10(5):593–619. doi: 10.1586/1744666X.2014.894886 24678812

[21] Santos JC de Brito CA Futata EA Azor MH Orii NM Maruta CW. Up-Regulation of Chemokine C-C Ligand 2 (CCL2) and C-X-C Chemokine 8 (CXCL8) Expression by Monocytes in Chronic Idiopathic Urticaria. Clin Exp Immunol (2012) 167(1):129–36. doi: 10.1111/j.1365-2249.2011.04485.x PMC3248094 22132892

[22] Moadab F Khorramdelazad H Abbasifard M. Role of CCL2/CCR2 Axis in the Immunopathogenesis of Rheumatoid Arthritis: Latest Evidence and Therapeutic Approaches. Life Sci (2021) 269:119034. doi: 10.1016/j.lfs.2021.119034 33453247

[23] Fuschiotti P. Role of IL-13 in Systemic Sclerosis. Cytokine (2011) 56(3):544–9. doi: 10.1016/j.cyto.2011.08.030 21920770

[24] Schmitz J Owyang A Oldham E Song Y Murphy E McClanahan TK. IL-33, an Interleukin-1-Like Cytokine That Signals *via* the IL-1 Receptor-Related Protein ST2 and Induces T Helper Type 2-Associated Cytokines. Immunity (2005) 23(5):479–90. doi: 10.1016/j.immuni.2005.09.015 16286016

[25] Riccardi A Borgia A Fasano S Messiniti V Iraace R Valentini G. Undifferentiated Connective Tissue Disease at Risk for SSc: Potential Role of Circulating CXCL-10, CXCL-11 and IL-33 in Predicting Disease Evolution. Arthritis Rheumatol (2019) 14(3):210–3.

[26] Valentini G Vettori S Cuomo G Iudici M D’Abrosca V Capocotta D. Early Systemic Sclerosis: Short-Term Disease Evolution and Factors Predicting the Development of New Manifestations of Organ Involvement. Arthritis Res Ther (2012) 14(4):R188. doi: 10.1186/ar4019 22901779 PMC3580584

[27] Valentini G Riccardi A Vettori S Irace R Iudici M Tolone S. CXCL4 in Undifferentiated Connective Tissue Disease at Risk for Systemic Sclerosis (SSc) (Previously Referred to as Very Early SSc). Clin Exp Med (2017) 17(3):411–4. doi: 10.1007/s10238-016-0437-y 27650429

[28] Gonzalez-Lopez L Rocha-Muñoz AD Olivas-Flores EM Garcia-Gonzalez A Peguero-Gómez AR Flores-Navarro J. Procollagen Type I and III Aminoterminal Propeptide Levels and Severity of Interstitial Lung Disease in Mexican Women With Progressive Systemic Sclerosis. Archivos Bronconeumología (English Edition) (2015) 51(9):440–8. doi: 10.1016/j.arbr.2014.06.027 25301411

[29] Witkowska AM. On the Role of sIL-2r Measurements in Rheumatoid Arthritis and Cancers. Mediat Inflamm (2005) 2005(3):121–30. doi: 10.1155/MI.2005.121 PMC1526469 16106097

[30] Vandercappellen J van Damme J Struyf S. The Role of the CXC Chemokines Platelet Factor-4 (CXCL4/PF-4) and its Variant (CXCL4L1/PF-4var) in Inflammation, Angiogenesis and Cancer. Cytokine Growth Factor Rev (2011) 22(1):1–18. doi: 10.1016/j.cytogfr.2010.10.011 21111666

[31] Romagnani P Maggi L Mazzinghi B Cosmi L Lasagni L Liotta F. CXCR3-Mediated Opposite Effects of CXCL10 and CXCL4 on T1 or T2 Cytokine Production. J Allergy Clin Immunol (2005) 116(6):1372–9. doi: 10.1016/j.jaci.2005.09.035 16337473

[32] Valentini G Riccardi A Vettori S Irace R Ludici M Tolone S. Serum CXCL4 Increase in Patients With Undifferentiated Connective Tissue Disease at Risk for Systemic Sclerosis Is Associated With Anti-Scl70 Antibodies and ICAM-1, a Marker of Endothelial Activation. Arthritis Rheumatol (2015) 67:3606–7.

[33] van Bon L Affandi AJ Broen J Christmann RB Marijnissen RJ Stawski L. Proteome-Wide Analysis and CXCL4 as a Biomarker in Systemic Sclerosis. New Engl J Med (2014) 370(5):433–43. doi: 10.1056/NEJMc1402401 PMC4040466 24350901

[34] Higgs BW Liu Z White B Zhu W White WI Morehouse C. Patients With Systemic Lupus Erythematosus, Myositis, Rheumatoid Arthritis and Scleroderma Share Activation of a Common Type I Interferon Pathway. Ann Rheum Dis (2011) 70(11):2029–36. doi: 10.1136/ard.2011.150326 21803750

[35] Tan FK Zhou X Mayes MD Gourh P Guo X Marcum C. Signatures of Differentially Regulated Interferon Gene Expression and Vasculotrophism in the Peripheral Blood Cells of Systemic Sclerosis Patients. Rheumatology (2006) 45(6):694–702. doi: 10.1093/rheumatology/kei244 16418202

[36] Brkic Z van Bon L Cossu M van Helden-Meeuwsen CG Vonk MC Knaapen H. The Interferon Type I Signature is Present in Systemic Sclerosis Before Overt Fibrosis and Might Contribute to its Pathogenesis Through High BAFF Gene Expression and High Collagen Synthesis. Ann Rheum Dis (2016) 75(8):1567–73. doi: 10.1136/annrheumdis-2015-207392 26371289

[37] Skaug B Assassi S. Type I Interferon Dysregulation in Systemic Sclerosis. Cytokine (2020) 132:154635. doi: 10.1016/j.cyto.2018.12.018 30685202

[38] Cossu M Andracco R Santaniello A Marchini M Severino A Caronni M. Serum Levels of Vascular Dysfunction Markers Reflect Disease Severity and Stage in Systemic Sclerosis Patients. Rheumatology (2016) 55(6):1112–6. doi: 10.1093/rheumatology/kew017 26989111

[39] Michalska-Jakubus M Kowal-Bielecka O Chodorowska G Bielecki M Krasowska D. Angiopoietins-1 and -2 are Differentially Expressed in the Sera of Patients With Systemic Sclerosis: High Angiopoietin-2 Levels are Associated With Greater Severity and Higher Activity of the Disease. Rheumatology (2011) 50(4):746–55. doi: 10.1093/rheumatology/keq392 21149250

[40] Tabata K Mikita N Yasutake M Matsumiya R Tanaka K Tani S. Up-Regulation of IGF-1, RANTES and VEGF in Patients With Anti-Centromere Antibody-Positive Early/Mild Systemic Sclerosis. Modern Rheumatol (2021) 31(1):171–6. doi: 10.1080/14397595.2020.1726599 32013651

[41] Ho YY Lagares D Tager AM Kapoor M. Fibrosis—a Lethal Component of Systemic Sclerosis. Nat Rev Rheumatol (2014) 10(7):390–402. doi: 10.1038/nrrheum.2014.53 24752182

[42] Cossu M van Bon L Preti C Rossato M Beretta L Radstake TRDJ. Earliest Phase of Systemic Sclerosis Typified by Increased Levels of Inflammatory Proteins in the Serum. Arthritis Rheumatol (2017) 69(12):2359–69. doi: 10.1002/art.40243 28859262

[43] Lacotte S Brun S Muller S Dumortier H. CXCR3, Inflammation, and Autoimmune Diseases. Ann NY Acad Sci (2009) 1173(1):310–7. doi: 10.1111/j.1749-6632.2009.04813.x 19758167

[44] Kuo PT Zeng Z Salim N Mattarollo S Wells JW Leggatt GR. The Role of CXCR3 and Its Chemokine Ligands in Skin Disease and Cancer. Front Med (Lausanne) (2018) 5:271. doi: 10.3389/fmed.2018.00271 30320116 PMC6167486

[45] Bellando Randone S George J Mazzotta C Guiducci S Furst DE Mor A. Angiostatic and Angiogenic Chemokines in Systemic Sclerosis: An Overview. J Scleroderma Related Disord (2017) 2(1):1–10. doi: 10.5301/jsrd.5000226

[46] Crescioli C Corinaldesi C Riccieri V Raparelli V Vasile M del Galdo F. Association of Circulating CXCL10 and CXCL11 With Systemic Sclerosis. Ann Rheum Dis (2018) 77(12):1845–6. doi: 10.1136/annrheumdis-2018-213257 PMC6241615 29760155

[47] Liu X Mayes MD Tan FK Wu M Reveille JD Harper BE. Correlation of Interferon-Inducible Chemokine Plasma Levels With Disease Severity in Systemic Sclerosis. Arthritis Rheumat (2013) 65(1):226–35. doi: 10.1002/art.37742 PMC3687352 23055137

[48] Ye L-L Wei X-S Zhang M Niu Y-R Zhou Q. The Significance of Tumor Necrosis Factor Receptor Type II in CD8+ Regulatory T Cells and CD8+ Effector T Cells. Front Immunol (2018) 9:583. doi: 10.3389/fimmu.2018.00583 29623079 PMC5874323

[49] Hügle T O’Reilly S Simpson R Kraaij MD Bigley V Collin M. Tumor Necrosis Factor-Costimulated T Lymphocytes From Patients With Systemic Sclerosis Trigger Collagen Production in Fibroblasts. Arthritis Rheumatol (2013) 65(2):481–91. doi: 10.1002/art.37738 PMC6588536 23045159

[50] Lee CG da Silva CA dela Cruz CS Ahangari F Ma B Kang M-J. Role of Chitin and Chitinase/Chitinase-Like Proteins in Inflammation, Tissue Remodeling, and Injury. Annu Rev Physiol (2011) 73:479–501. doi: 10.1146/annurev-physiol-012110-142250 21054166 PMC3864643

[51] la Montagna G D’Angelo S Valentini G. Cross-Sectional Evaluation of YKL-40 Serum Concentrations in Patients With Systemic Sclerosis. Relationship with Clinical and Serological Aspects of Disease. J Rheumatol (2003) 30(10):2147–51. 14528508

[52] Cossu M van Bon L Nierkens S Bellocchi C Santaniello A Dolstra H. The Magnitude of Cytokine Production by Stimulated CD56+ Cells is Associated With Early Stages of Systemic Sclerosis. Clin Immunol (2016) 173:76–80. doi: 10.1016/j.clim.2016.09.004 27616458

[53] Solomon DH Kavanaugh AJ Schur PH. American College of Rheumatology *Ad Hoc* Committee on Immunologic Testing Guidelines. Evidence-Based Guidelines for the Use of Immunologic Tests: Antinuclear Antibody Testing. Arthritis Rheumatol (2002) 47(4):434–44. doi: 10.1002/art.10561 12209492

[54] Orme ME Voreck A Aksouh R Ramsey-Goldman R Schreurs MWJ. Systematic Review of anti-dsDNA Testing for Systemic Lupus Erythematosus: A Meta-Analysis of the Diagnostic Test Specificity of an anti-dsDNA Fluorescence Enzyme Immunoassay. Autoimmun Rev (2021) 20(11):102943. doi: 10.1016/j.autrev.2021.102943 34508916

[55] Flechsig A Rose T Barkhudarova F Strauss R Klotsche J Dähnrich C. What is the Clinical Significance of Anti-Sm Antibodies in Systemic Lupus Erythematosus? A Comparison With anti-dsDNA Antibodies and C3. Clin Exp Rheumatol (2017) 35(4):598–606. 28281463

[56] Slater CA Davis RB Shmerling RH. Antinuclear Antibody Testing. A study of clinical utility. Arch Intern Med (1996) 156(13):1421–5. doi: 10.1001/archinte.156.13.1421 8678710

[57] Munroe ME Young KA Kamen DL Guthridge JM Niewold TB Costenbader KH. Discerning Risk of Disease Transition in Relatives of Systemic Lupus Erythematosus Patients Utilizing Soluble Mediators and Clinical Features. Arthritis Rheumatol (2017) 69(3):630–42. doi: 10.1002/art.40004 PMC5329053 27863174

[58] Hallengren CS Nived O Sturfelt G. Outcome of Incomplete Systemic Lupus Erythematosus After 10 Years. Lupus (2004) 13(2):85–8. doi: 10.1191/0961203304lu477oa 14994999

[59] al Daabil M Massarotti EM Fine A Tsao H Ho P Schur PH. Development of SLE Among “Potential SLE” Patients Seen in Consultation: Long-Term Follow-Up. Int J Clin Pract (2014) 68(12):1508–13. doi: 10.1111/ijcp.12466 PMC4241393 24853089

[60] Vila L Mayor A Valent A Garc M Vila S. Clinical Outcome and Predictors of Disease Evolution in Patients With Incomplete Lupus Erythematosus. Lupus (2000) 9(2):110–5. doi: 10.1191/096120300678828073 10787007

[61] Eriksson C Rantapää-Dahlqvist S. Cytokines in Relation to Autoantibodies Before Onset of Symptoms for Systemic Lupus Erythematosus. Lupus (2014) 23(7):691–6. doi: 10.1177/0961203314523869 24531079

[62] Bezalel S Guri KM Elbirt D Asher I Sthoeger ZM. Type I Interferon Signature in Systemic Lupus Erythematosus. Isr Med Assoc J (2014) 16(4):246–9. 24834763

[63] Li Q-Z Zhou J Lian Y Zhang B Branch VK Carr-Johnson F. Interferon Signature Gene Expression is Correlated With Autoantibody Profiles in Patients With Incomplete Lupus Syndromes. Clin Exp Immunol (2010) 159(3):281–91. doi: 10.1111/j.1365-2249.2009.04057.x PMC2819494 19968664

[64] Oke V Gunnarsson I Dorschner J Eketjäll S Zickert A Niewold TB. And Type III Associate With Distinct Clinical Features of Active Systemic Lupus Erythematosus. Arthritis Res Ther (2019) 21(1):107. doi: 10.1186/s13075-019-1878-y 31036046 PMC6489203

[65] Schroder K Hertzog PJ Ravasi T Hume DA. Interferon-Gamma: An Overview of Signals, Mechanisms and Functions. J Leukoc Biol (2004) 75(2):163–89. doi: 10.1189/jlb.0603252 14525967

[66] Harigai M Kawamoto M Hara M Kubota T Kamatani N Miyasaka N. Excessive Production of IFN-Gamma in Patients With Systemic Lupus Erythematosus and its Contribution to Induction of B Lymphocyte Stimulator/B Cell-Activating Factor/TNF Ligand Superfamily-13B. J Immunol (2008) 181(3):2211–9. doi: 10.4049/jimmunol.181.3.2211 18641361

[67] Scapini P Nardelli B Nadali G Calzetti F Pizzolo G Montecucco C. G-CSF–stimulated Neutrophils Are a Prominent Source of Functional BLyS. J Exp Med (2003) 197(3):297–302. doi: 10.1084/jem.20021343 12566413 PMC2193843

[68] Mackay F Schneider P Rennert P Browning J. BAFF AND APRIL: A Tutorial on B Cell Survival. Annu Rev Immunol (2003) 21:231–64. doi: 10.1146/annurev.immunol.21.120601.141152 12427767

[69] Schneider P MacKay F Steiner V Hofmann K Bodmer JL Holler N. BAFF, a Novel Ligand of the Tumor Necrosis Factor Family, Stimulates B Cell Growth. J Exp Med (1999) 189(11):1747–56. doi: 10.1084/jem.189.11.1747 PMC2193079 10359578

[70] Munroe ME Lu R Zhao YD Fife DA Robertson JM Guthridge JM. Altered Type II Interferon Precedes Autoantibody Accrual and Elevated Type I Interferon Activity Prior to Systemic Lupus Erythematosus Classification. Ann Rheum Dis (2016) 75(11):2014–21. doi: 10.1136/annrheumdis-2015-208140 PMC4959992 27088255

[71] Lu R Munroe ME Guthridge JM Bean KM Fife DA Chen H. Dysregulation of Innate and Adaptive Serum Mediators Precedes Systemic Lupus Erythematosus Classification and Improves Prognostic Accuracy of Autoantibodies. J Autoimmun (2016) 74:182–93. doi: 10.1016/j.jaut.2016.06.001 PMC5079766 27338520

[72] Olsen NJ McAloose C Carter J Han BK Raman I Li Q-Z. Clinical and Immunologic Profiles in Incomplete Lupus Erythematosus and Improvement With Hydroxychloroquine Treatment. Autoimmune Dis (2016) 2016:1–9. doi: 10.1155/2016/8791629 PMC5225311 28116147

