# Supplementary material for: Precursors to Systemic Sclerosis and Systemic Lupus Erythematosus: From Undifferentiated Connective Tissue Disease to the Development of Identifiable Connective Tissue Diseases
Source: Front Immunol. 2022 May 5;13:869172. doi: 10.3389/fimmu.2022.869172 (PMC9118990; doi:10.3389/fimmu.2022.869172)
Supplement: Supplementary file 1 [file DataSheet_1.docx]

**Supplementary Information**

**1. Supplementary Data S1: Search Strategy Medline**

1     exp Lupus Erythematosus, Systemic/ (61824)

2     (libman$ sacks$ adj3 disease$).tw,kf. (52)

3     (lupus$ adj3 erythematos$ adj5 (disseminat$ or systemic$)).tw,kf. (56181)

4     (lupus$ adj3 (nephrit$ or glomerulo?nephrit$)).tw,kf. (9519)

5     ((CNS or central nervous system$ or meningoencephaliti$) adj3 lupus$).tw,kf. (390)

6     or/1-5 (78098)

7     exp scleroderma, systemic/ (21471)

8     ((scleroderm$ or schleroderm$ or dermato?scleros$ or scleros$) adj5 systemic$).tw,kf. (18614)

9     or/7-8 (26975)

10     6 or 9 (100574)

11     Undifferentiated Connective Tissue Diseases/ (112)

12     (undifferentiat$ and (connective$ adj5 tissue$) and disease$).mp. (692)

13     (undifferentiat$ and (connective$ adj3 disease$)).mp. (613)

14     UCTD$1.tw,kf. (234)

15     or/11-14 (742)

16     10 and 15 (313)

17     (precursor$ or (predisease$ or pre-disease$ or presymptomatic$ or pre-symptomatic$)).tw,kf. (260402)

18     ((prior to or before) adj3 onset$1 adj3 symptom$).tw,kf. (3267)

19     ((predate$ or early or predating$ or recent$) adj3 onset$1).tw,kf. (53990)

20     (pre-SSc or pre-SLE).tw,kf. (20)

21     (very early$ or (early adj3 (SSc or SLE))).tw,kf. (19896)

22     or/17-21 (334031)

23     10 and 22 (1656)

24     ((lupus$ adj3 (nephrit$ or glomerulo?nephrit$ or (erythematos$ adj5 (disseminat$ or systemic$)) or CNS or central nervous system$ or meningoencephaliti$)) or ((scleroderm$ or schleroderm$ or dermato?scleros$ or scleros$) adj5 systemic$)).ti. or ((lupus$ adj3 (nephrit$ or glomerulo?nephrit$ or (erythematos$ adj5 (disseminat$ or systemic$)) or CNS or central nervous system$ or meningoencephaliti$)) or ((scleroderm$ or schleroderm$ or dermato?scleros$ or scleros$) adj5 systemic$)).ab. /freq=5 (48384)

25     early$.ti. or early$.ab. /freq=4 (352035)

26     (patient$ and (prognos$ or predict$ or progres$ or detect$ or diagnos$ or patho$)).tw,kf. (3336195)

27     24 and 25 and 26 (428)

28     10 and 27 (428)

29     16 or 23 or 28 (2201)

30     29 not (exp Animals/ not (Human/ and exp Animals/)) (2042)

31     (animal$1 or mice or rat or rats or cat$1 or cattle$1 or dog$1 or goat$1 or horse$1 or rabbit$1 or sheep$1 or swine$1 or pig$1 or piglet* or canine$1 or feline$1 or porcine$ or calf or primate* or rodent$ or hamster$ or lamb$1 or monkey$1 or murine or veterinar*).ti. (2155621)

32     30 not 31 (2024)

33     exp case-control studies/ or (case$ and control$).tw,kf. or (case$ and series).tw,kf. (1738018)

34     case reports/ or case report$.mp. (2292225)

35     32 not (34 not (33 and 34)) (1776)

36     35 not case report.ti. (1772)

37 limit 36 to yr=2010-Current” (1030)

**2. Supplementary Data S2: Search Strategy for Embase**

1     exp *systemic lupus erythematosus/ (62874)

2     (libman$ sacks$ adj3 disease$).tw,kw. (44)

3     (lupus$ adj3 erythematos$ adj5 (disseminat$ or systemic$)).tw,kw. (82302)

4     (lupus$ adj3 (nephrit$ or glomerulo?nephrit$)).tw,kw. (16160)

5     ((CNS or central nervous system$ or meningoencephaliti$) adj3 lupus$).tw,kw. (585)

6     or/1-5 (101539)

7     exp *systemic sclerosis/ (23393)

8     ((scleroderm$ or schleroderm$ or dermato?scleros$ or scleros$) adj5 systemic$).tw,kw. (31779)

9     or/7-8 (35310)

10     6 or 9 (131687)

11     undifferentiated connective tissue disease/ (212)

12     (undifferentiat$ and (connective$ adj5 tissue$) and disease$).tw,kw. (1154)

13     (undifferentiat$ and (connective$ adj3 disease$)).tw,kw. (1034)

14     UCTD$1.tw,kw. (544)

15     or/11-14 (1381)

16     10 and 15 (634)

17     precursor/ or precursor$.tw,kw. or (predisease$ or pre-disease$ or presymptomatic$ or pre-symptomatic$).ti. or (predisease$ or pre-disease$ or presymptomatic$ or pre-symptomatic$).ab. /freq=2 (311819)

18     ((prior to or before) adj3 onset$1 adj3 symptom$).tw,kw. (5514)

19     ((predate$ or early or predating$ or recent$) adj3 onset$1).tw,kw. (80350)

20     (pre-SSc or pre-SLE).tw,kw. (44)

21     very early$.tw,kw. or (early adj2 (SSc or SLE)).ti. or (early adj2 (SSc or SLE)).ab. /freq=2 (28788)

22     or/17-21 (421616)

23     10 and 22 (1943)

24     ((lupus$ adj3 (nephrit$ or glomerulo?nephrit$ or (erythematos$ adj5 (disseminat$ or systemic$)) or CNS or central nervous system$ or meningoencephaliti$)) or ((scleroderm$ or schleroderm$ or dermato?scleros$ or scleros$) adj5 systemic$)).ti. or ((lupus$ adj3 (nephrit$ or glomerulo?nephrit$ or (erythematos$ adj5 (disseminat$ or systemic$)) or CNS or central nervous system$ or meningoencephaliti$)) or ((scleroderm$ or schleroderm$ or dermato?scleros$ or scleros$) adj5 systemic$)).ab. /freq=5 (71528)

25     early$.ti. or early$.ab. /freq=4 (477376)

26     (patient$ and (prognos$ or predict$ or progres$ or detect$ or diagnos$ or patho$)).tw,kw. (5393079)

27     24 and 25 and 26 (947)

28     10 and 27 (947)

29     16 or 23 or 28 (3214)

30     29 not ((exp animal/ or nonhuman/) not exp human/) (3040)

31     (animal$1 or mice or rat or rats or cat$1 or cattle$1 or dog$1 or goat$1 or horse$1 or rabbit$1 or sheep$1 or swine$1 or pig$1 or piglet* or canine$1 or feline$1 or porcine$ or calf or primate* or rodent$ or hamster$ or lamb$1 or monkey$1 or murine or veterinar*).ti. (2623110)

32     30 not 31 (3023)

33     exp case control study/ or (case$ and control$).tw,kw. or exp case study/ or (case$ and series).tw,kw.  (1192697)

34     case report/ or case report$.tw,kw. (2833351)

35     32 not (34 not (33 and 34)) (2724)

36     35 not case report.ti. (2718)

37 limit 36 to yr=”2010-Current” (2044)
